# Lymph vascular invasion in invasive mammary carcinomas identified by the endothelial lymphatic marker D2-40 is associated with other indicators of poor prognosis

**DOI:** 10.1186/1471-2407-8-64

**Published:** 2008-02-29

**Authors:** Vanessa FZ Marinho, Konradin Metze, Fernanda SF Sanches, Gislene FS Rocha, Helenice Gobbi

**Affiliations:** 1 Departamento de Anatomia Patológica, Faculdade de Medicina da Universidade Federal de Minas Gerais (UFMG), Belo Horizonte, Brazil; 2 Interdisciplinary group: "Analytical Cellular Pathology", Departamento de Patologia, Universidade Estadual de Campinas (UNICAMP), Campinas, Brazil

## Abstract

**Background:**

Immunohistochemical studies of lymphatic vessels have been limited by a lack of specific markers. Recently, the novel D2-40 antibody, which selectively marks endothelium of lymphatic vessels, was released. The aim of our study is to compare lymphatic and blood vessel invasion detected by hematoxylin and eosin (H&E) versus that detected by immunohistochemistry, relating them with morphologic and molecular prognostic factors.

**Methods:**

We selected 123 cases of invasive mammary carcinomas stratified into three subgroups according to axillary lymph node status: macrometastases, micrometastases, and lymph node negative. Lymphatic (LVI) and blood (BVI) vessel invasion were evaluated by H&E and immunohistochemistry using the D2-40 and CD31 antibodies, and related to histologic tumor type and grade, estrogen and progesterone receptors, E-cadherin, Ki67, p53, and Her2/*neu *expression.

**Results:**

LVI was detected in H&E-stained sections in 17/123 cases (13.8%), and in D2-40 sections in 35/123 cases (28.5%) (Kappa = 0.433). BVI was detected in H&E-stained sections in 5/123 cases (4.1%), and in CD31 stained sections in 19/123 cases (15.4%) (Kappa = 0.198). LVI is positively related to higher histologic grade (p = 0.013), higher Ki67 expression (p = 0.00013), and to the presence of macrometastases (p = 0.002), and inversely related to estrogen (p = 0.0016) and progesterone (p = 0.00017) receptors expression.

**Conclusion:**

D2-40 is a reliable marker of lymphatic vessels and is a useful tool for lymphatic emboli identification in immunostained sections of breast carcinomas with higher identification rates than H&E. Lymphatic vessel invasion was related to other features (high combined histologic grade, high Ki67 score, negative hormone receptors expression) associated with worse prognosis, probable reflecting a potential for lymphatic metastatic spread and aggressive behavior.

## Background

Lymphatic vessels are considered the main route by which tumor cells reach axillary lymph nodes [1-3]. Lymphatic vessel invasion (LVI) is known as an independent predictor of lymph node metastases in breast cancer. The diagnosis of LVI is made based on the presence of tumor emboli within vascular channels lined by a single layer of endothelial cells without red blood cells. Lymphatic vessels are flattened channels or open spaces lined by a single layer of endothelial cells whose lumen are sometimes filled with lymphocytes. However, the identification of LVI is difficult in hematoxylin and eosin (H&E) stained slides. Retraction artifacts that isolate tumor aggregates due to tissue shrinkage during fixation are sometimes confused with the true tumor emboli in lymphatic vessels [4,5].

Several markers of endothelial cells have been used, including CD31, CD34, and factor VIII-related antigen. However, studies of lymphatic vessels have been limited by lack of specific lymphatic endothelial markers and immunohistochemical identification of lymphatic vessels has been unreliable [1,6]. Novel selective markers for lymphatic endothelium have been released, such as LYVE-1, Prox-1, desmoplakin, and podoplanin [7-12]. More recently, the monoclonal antibody D2-40 was shown to selectively detect lymphatic vessels in breast and tonsillar tissue [13,14]. D2-40 is an IgG2a monoclonal antibody that was generated against an oncofetal antigen M2A, which is normally expressed in the fetal testis and reexpressed in germ cell neoplasia [15]. It is a novel monoclonal antibody to an M_r _40000 O-linked sialoglycoprotein that reacts with a fixation-resistant epitope on the lymphatic endothelium [16] The D2-40 antibody has been shown to specifically recognize podoplanin, a glomerular podocyte membrane protein [17,18] and has been shown to be a very sensitive and specific marker for lymphatic endothelium in most tissues [19] and especially in breast cancer [20]. D2-40 stains the endothelium of lymphatic vessels, lymphangiomas, Kaposi's sarcoma and Dabska tumor, but does not stain endothelium of blood vessels, hemangiomas, glomus tumors, angiolipomas, pyogenic granulomas, and vascular malformations [13,14,21,22].

The aim of our study is to compare lymphatic vessel invasion (LVI) and blood vessel invasion (BVI) in invasive mammary carcinomas using H&E and immunohistochemical stained sections, relating them to other prognostic factors.

## Methods

We selected 123 patients with invasive mammary carcinomas (IMC), who had been submitted to surgical treatment with axillary lymph node dissection at our hospital between 1990 and 2004. The research was approved by the Ethics Committee of the University. On original histopathologic examination, 41 cases were diagnosed as axillary lymph node negative, 41 cases with micrometastases (defined as neoplastic cell clusters measuring between 0.2 and 2 mm), and 41 cases with macrometastases (defined as neoplastic cell clusters larger than 2 mm) [23]. No case of isolated tumor cells was included in this study. The mean age of patients, the histologic type and tumor grade of cases of each subgroup were similar. For all cases, the original H&E-stained sections of primary tumors and axillary lymph nodes were available for histologic review. The paraffin blocks were available for additional sections and immunohistochemical analysis. Special care was taken to include only specimens with sufficient amount of normal tissue surrounding the invasive tumor to evaluate peritumoral LVI and BVI.

Cases were evaluated with regard to age of patients and tumor size using the TNM system [23]. Histologic analysis of primary breast carcinoma features included histologic type, histologic grade, LVI and BVI. The histologic type of primary tumor was classified based on Page *et al *[24], and the College of American Pathologists recommendations [25], using rigid criteria for classification of special types. Tumor grade was determined using the Nottingham grading system [26]. The diameter of the microscopic field used for mitotic count was 0.44 mm. Primary tumors were analyzed without acknowledgment of the lymph node status.

H&E-stained sections of primary tumors were reviewed and new histologic consecutive, serial sections were prepared for immunohistochemical analysis. Immunohistochemistry (IHC) was performed on 5 μm thickness sections, using monoclonal antibodies (Table 1) and the streptavidin-biotin peroxidase method. Heat-induced epitope retrieval was done with citrate buffer pH6.0 for 25 minutes. Immunohistochemical reactions were developed with diaminobenzidine and sections counterstained with Harrris hematoxylin. All immunostains were manually processed.

**Table 1:** Primary antibodies, dilutions, antigen retrieval, and sources of antibodies used in the immunohistochemical study

ANTIBODY	CLONE	DILUTION	ANTIGEN RETRIEVAL	SOURCE/COUNTRY
D2-40	D2-40	1:100	No	Signet/USA
CD31	JC/70A	1:50	Yes	Dako/USA
P53	DO7	1:400	Yes	Dako/USA
ER	6F-11	1:100	Yes	Novocastra/UK
PR	PgR 312	1:100	Yes	Novocastra/UK
Ki67	MIB-1	1:50	Yes	Immunotech/France
Her2/*neu*	CB11	1:80	No	Novocastra/UK
E-cadherin	NCH-38	1:50	Yes	Dako/USA
Cytokeratin	AE1/AE3	1:100	Yes	Dako/USA

IHC using monoclonal anti-cytokeratin antibody (clone AE1/AE3, Dako, USA) was carried out on sections of axillary lymph nodes classified as negative to confirm this status. Cytokeratin reactions were scored as negative or positive. All axillary lymph nodes initially classified as negative continued negative after IHC staining (Table 1).

The IHC-stained sections of all primary tumors and axillary lymph nodes were evaluated without knowledge of previous node status. All positive lymph nodes, doubts, and 10% of negative nodes were re-evaluated by two pathologists (VFZM and HG) using a double-headed microscope. LVI and/or BVI were assessed by the same pathologists reviewing the H&E and immunohistochemical stained sections. In H&E-stained sections, LVI and/or BVI were considered evident if at least one tumor cell cluster was clearly visible in the vascular space. In this study, we defined LVI in H&E-stained sections, as tumor cell nests in spaces and around the clump of tumor cell nests that were lined by flattened endothelium with no supporting smooth muscle or elastica, and/or were filled with lymphatic fluid. Tumor cell nests in spaces that were either not lined by endothelial cells or were lined by endothelial-like cells, probably tumor-stromal fibroblasts, were classified as stroma-invasive tumor cell nests. Similarly, we defined BVI in H&E-stained sections, as tumor cell nests in spaces and around the clump of tumor cell nests that were lined by endothelium, that is, not flattened, and/or were filled with red blood cells [16]. The whole section was examined. In order to score lymphatic invasion, only peritumoral areas, and peritumoral lymphatic invasion were counted as lymphatic invasion. Intratumoral lymphatic invasion was not counted in this study. In most cases, intratumoral lymphatic invasion can be misinterpreted as retraction artifact and it was not the purpose of our study to evaluate artifacts.

**Figure 1:**
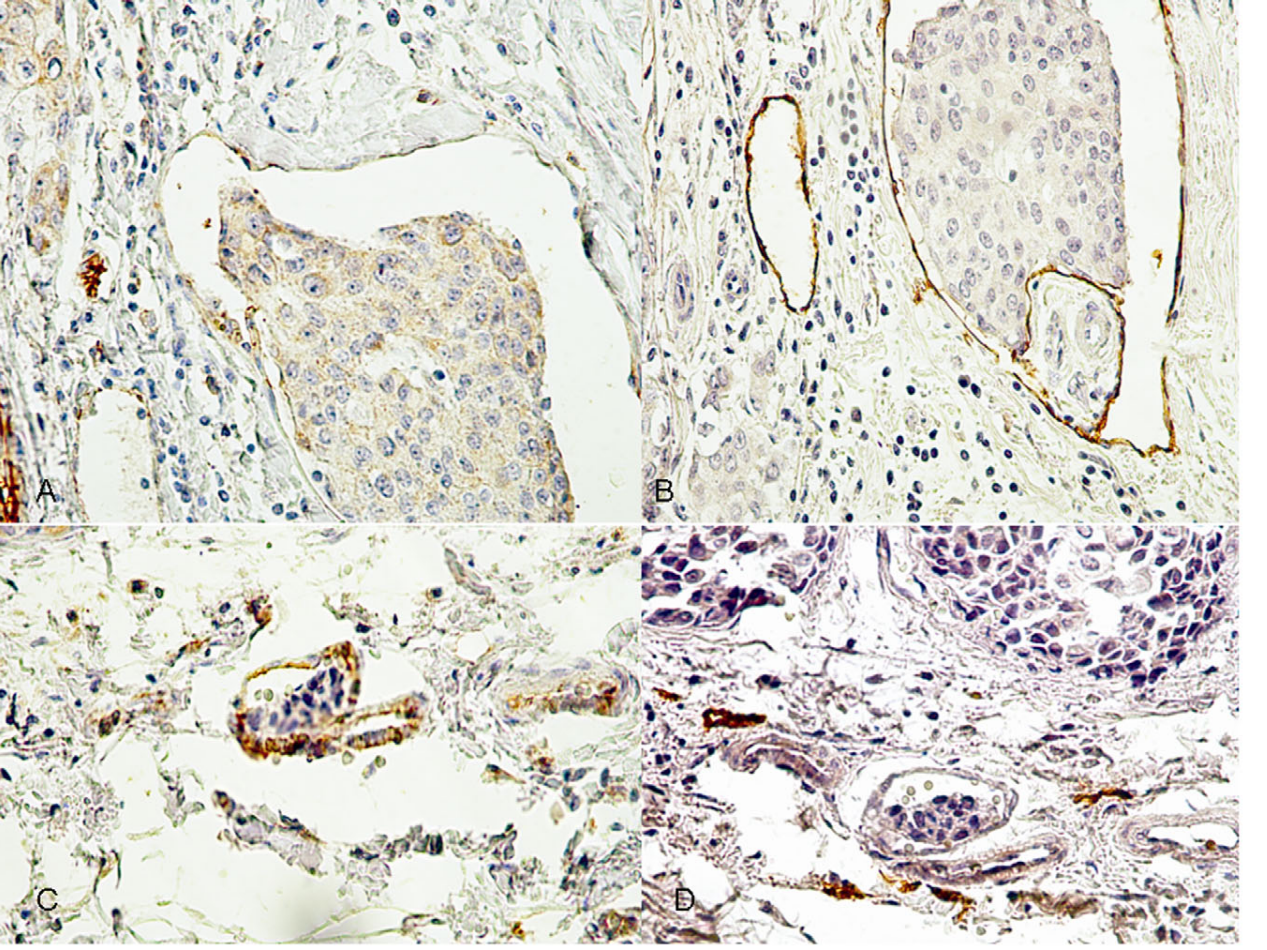
**Lymphatic and blood vessels in sections of invasive breast cancer stained for D2-40 and CD31**. Lymphatic vessel invasion stained for CD31(A) and D2-40 (B) in sections of breast tumors from the same case. In A, CD31 positive endothelium and in B, D2-40 positive endothelium (×400). Blood vessel invasion stained for CD31 (C) and D2-40 (D) in sections of breast tumors from the same case. In C, CD31 positive endothelium and in D, D2-40 negative endothelium (×400). In B and C, the endothelium of lymphatic vessels without invasion was also immunostained.

For this analysis, we evaluated LVI in adjacent stromal tissue (peritumoral area) with a ×200 magnification, with sequential assessment by two investigators, as recommended by the First International Consensus on the Methodology of Lymphangiogenesis Quantification in Solid Human Tumors [27]. We have not used double immunostaining for D2-40 and Ki67 monoclonal antibody for the purpose of counting the number of proliferating lymphatic endothelial cells [27]. Vessels were considered as lymphatic when the endothelium stained with both D2-40 and CD31 antibodies. Vessels were classified as blood vessels when the endothelium stained only for the CD31 monoclonal antibody and was negative for D2-40 in consecutive sections. Vessels that were D2-40 and CD31 positive and had red blood cells in the lumen were considered lymphatic vessels. The red blood cells could be present in lymphatic-blood vessel capillary shunting or bleeding into lymphatic vessels. Also, vessels that were D2-40 positive but were negative for CD31 antibody were considered lymphatic vessels (Figure 1).

Estrogen (ER) and progesterone (PR) receptor expression were determined by a method for scoring immunostaining signal, according to Allred *et al *[28,29]. A proportion score (PS) is assigned representing the estimated proportion of tumor cells staining considering all positive cells (range 0–5). Score 0 refers to no cells having been stained; score 1, 1% of tumor cells stained; score 2, 10% of cells stained; score 3, 1/3 of cells stained; score 4, 2/3 of cells stained; and score 5, 100% of cells stained. An intensity score (IS) is assigned representing the estimated average staining intensity of positive tumor cells (range 0–3). A total score (TS) is calculated from the sum of PS and IS (ranging from 0, 2–8) [28]. A positive result for both ER and PR is defined as TS ≥ 3, which was validated in clinical studies [28,29]. Ki67 was evaluated by the percentage of neoplastic cells showing nuclear staining in the most proliferative area ("hot spot"). Tumors were grouped in four categories: <10%, 10–25%, 25–50% and >50% [30]. Cases were classified as p53 positive when more than 10% of neoplastic cells exhibited positive nuclear staining [25]. The Her2/*neu *stained sections were classified according to Dako-HercepTest™ scoring system [31]. E-cadherin (E-cad) reactivity was analyzed using a semiquantitative scoring system and only the citoplasmic membrane staining was considered. The reactivity was graded as "0" if there was no evidence of staining, "1+" if 1–25% of cells stained, "2+" if 26–49% stained, "3+" if 50–74% stained, and "4+" if >75% of cells stained [32].

Statistical analysis was performed using the Epi-info 6.04b, SPSS 8.0 and WinStat software. We calculated McNemars sign test and Cohen's Kappa for concordance regarding vessel invasion evaluated with different methods and the correlation coefficients between variables according to Pearson or Spearman. Comparisons among the three groups were done by one-way analysis of variance (ANOVA) or Kruskall-Wallis test. The relationship between LVI and BVI and tumor characteristics was evaluated with the help of a logistic regression analysis [33,34].

## Results

The age of patients ranged from 27 to 88 years (mean = 55.9 years, median = 52 years). Clinicopathologic features of the tumors are summarized in Table 2.

**Table 2:** Clinicopathologic features of 123 cases of invasive mammary carcinomas according to axillary lymph node status

**Characteristics**	**Mac-Met n (%)**	**Mic-Met n (%)**	**LNN n (%)**	**Total n (%)**
**Age**				
≤ 52 years	22 (53.7)	21 (51.2)	20 (48.8)	63 (51.2)
>52 years	19 (46.3)	20 (49.8)	21 (51.2)	60 (49.8)
**Menopausal status**				
Premenopausal	20 (48.8)	18 (43.9)	20 (48.8)	58 (47.2)
Postmenopausal	21 (51.2)	23 (56.1)	21 (51.2)	65 (52.8)
**Tumor Size (TNM)**				
T1	10 (24.4)	17 (41.5)	17 (41.5)	44 (35.8)
T2	24 (58.5)	23 (56.1)	18 (43.9)	65 (52.8)
T3	7 (17.1)	1 (2.4)	6 (14.6)	14 (11.4)
**Histologic type**				
Ductal NST	36 (87.8)	33 (80.4)	36 (87.8)	105 (85.4)
Lobular	3 (7.4)	5 (12.2)	2 (4.8)	10 (8.1)
Other type	2 (4.8)	3 (7.4)	3 (7.4)	8 (6.5)
Histologic grade*				
Grade I	9 (22)	14 (34.1)	13 (31.7)	36 (29.3)
Grade II	21 (51.2)	18 (43.9)	19 (46.3)	58 (47.2)
Grade III	11 (26.8)	9 (22)	9 (22)	29 (23.6)
**Total**	41 (100)	41 (100)	41 (100)	123 (100)

Mac-Met = macrometastases; Mic-Met = micrometastases; LNN = lymph node negative; n = number of cases; NST = non-special type

*Tumors with lymphatic invasion showed a higher histologic grade (p = 0.013). This correlation with lymphatic invasion was significant in H&E-sections and also in IHC-sections.

Immunohistochemical features according to axillary lymph node status are summarized in Table 3.

**Table 3:** Immunohistochemical features of 123 cases of invasive mammary carcinomas according to axillary lymph node status

**IHC features**	**Mac-Met n (%)**	**Mic-Met n (%)**	**LNN n (%)**	**Total n (%)**
ER *				
Positive	31 (75.6)	35 (85.4)	34 (82.9)	100 (81.3)
Negative	10 (24.4)	6 (14.6)	7 (17.1)	23 (18.7)
PR *				
Positive	25 (61)	29 (70.7)	28 (68.3)	82 (66.7)
Negative	16 (39)	12 (29.3)	13 (31.7)	41 (33.3)
**Her2/*neu* score**				
0 and 1+	35 (85.4)	32 (78)	36 (87.8)	103 (83.7)
2+	1 (2.4)	1 (2.4)	1 (2.4)	3 (2.4)
3+	5 (12.2)	8 (19.5)	4 (9.8)	17 (13.8)
**P53**				
Positive	17 (41.5)	11 (26.8)	10 (24.4)	38 (30.9)
Negative	24 (58.5)	30 (73.2)	31 (75.6)	85 (69.1)
Ki67 score *				
<10%	16 (39)	23 (56.1)	16 (39)	55 (44.7)
10–25%	11 (26.8)	9 (22)	13 (31.7)	33 (26.8)
25–50%	10 (24.4)	6 (14.6)	8 (19.5)	24 (19.5)
>50%	4 (9.8)	3 (7.3)	4 (9.8)	11 (8.9)
**E-cadherin score**				
0 (negative)	2 (4.9)	1 (2.4)	2 (4.9)	5 (4.1)
1+	3 (7.3)	3 (7.3)	2 (4.9)	8 (6.5)
2+	0 (0)	2 (4.9)	1 (2.4)	3 (2.4)
3+	8 (19.5)	6 (14.6)	4 (9.8)	18 (14.6)
4+	28 (68.3)	29 (70.7)	32 (78)	89 (72.4)
**Total**	41 (100)	41 (100)	41 (100)	123 (100)

Mac-Met = macrometastases; Mic-Met = micrometastases; LNN = lymph node negative; n = number of cases; IHC = immunohistochemical; ER = estrogen receptor; PR = progesterone receptor

* Tumors with lymphatic invasion showed an increased Ki67 score (p = 0.00013), but lower estrogen (p = 0.0016) and progesterone (p = 0.00017) receptor scores. This correlation with lymphatic invasion was significant in H&E-sections and also in IHC-sections.

The other variables showed no statistical significant correlations with LVI identified either by H&E or IHC-sections.

**Figure 2:**
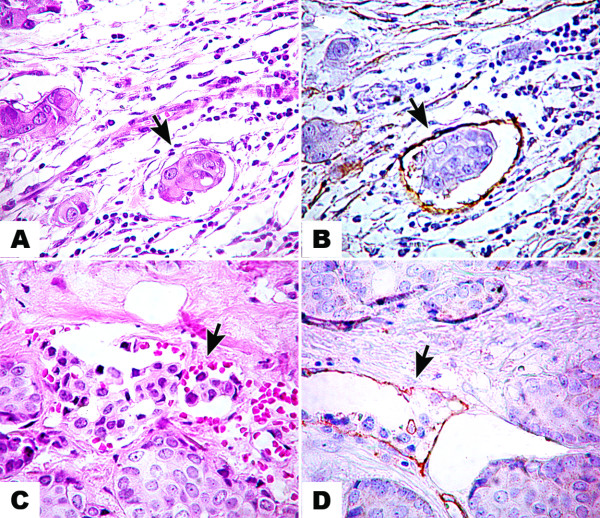
**Lymphatic and blood vascular invasion in invasive breast cancer in H&E and immunostained slides**. Lymphatic vascular invasion (arrow) seen in H&E (A) and D2-40 (B) stained sections of breast tumors from the same case; ×400; Blood vessel invasion (arrow) seen in H&E (C) and CD31 (D) stained sections; ×400.

LVI and BVI were found more frequently in the immunostained sections than the H&E-stained sections (Table 4; Figure 2). We detected LVI in 38/123 cases (30.9%) and BVI in 21/123 cases (17.1%), when adding the two detection methods. In 11/123 cases we detected BVI and LVI in the same case. In 10/123 cases we detected only BVI and in 27/123 cases only LVI. Cohen's Kappa for the diagnostic agreement between both methods was moderate for lymphatic vessel invasion (Kappa = 0.433) and poor for blood vessel invasion (Kappa = 0.198) (Table 4). Table 4 includes three cases of LVI detected in H&E-stained slides that were not found in IHC sections. The foci of LVI in these cases were extremely small and a single focus was present in each case. These extremely small foci were not present on the deeper sections used for IHC. The same situation occurred in two cases of BVI (Table 4). In our cases, we did not detect emboli in sections that were D2-40 positive and consecutively CD31 negative. All cases that showed red blood cells inside the lumen and tumor emboli, were D2-40 negative and CD31 positive, and these vessels were considered blood vessels.

**Table 4:** Lymphatic and blood vessel invasion detected on H&E and immunostained sections of 123 invasive mammary carcinomas

**H&E**	**Immunohistochemistry**		
	
	**Positive n (%)**	**Negative n (%)**	**Total n (%)**
**LVI**			
**Positive**	14 (11.4)	3 (2.4)	17 (13.8)
**Negative**	21 (17.1)	85 (69.1)	106 (86.2)
**Total**	35 (28.5)	88 (71.5)	123 (100)
**BVI**			
**Positive**	3 (2.4)	2 (1.7)	5 (4.1)
**Negative**	16 (13.0)	102 (82.9)	118 (95.9)
**Total**	19 (15.4)	104 (84.6)	123 (100)

LVI = lymphatic vessel invasion; BVI = blood vessel invasion; H&E = hematoxylin and eosin; n = number of cases

Kappa value for LVI= 0.433 (95% confidence interval: 0.229 to 0.636)

Kappa value for BVI= 0.198 (95% confidence interval: -0.144 to 0.541)

Results of the LVI and BVI assessed by H&E and immunohistochemistry are summarized in Table 5.

**Table 5:** Lymphatic and blood vessel invasion in 123 invasive mammary carcinomas stratified according to nodal status

	**Mac-Met n (%)**	**Mic-Met n (%)**	**LNN n (%)**	**Total n (%)**
LVI (H&E) *	12 (29.3)	3 (7.3)	2 (4.9)	17 (13.8)
**LVI (IHC)**	16 (39)	10 (24.4)	9 (22)	35 (28.5)
**BVI (H&E)**	2 (4.9)	1 (2.4)	2 (4.9)	5 (4.1)
**BVI (IHC)**	6 (14.6)	11 (26.8)	2 (4.9)	19 (15.4)

Mac-Met = macrometastases; Mic-Met = micrometastases; LNN = lymph node negative; LVI = lymphatic vessel invasion; BVI = blood vessel invasion; n = number of cases; H&E = hematoxylin and eosin; IHC = immunohistochemistry

* p value = 0.002. The other p values were not statistically significant.

LVI was positively related to the presence of metastases (p = 0.002) (Table 5), and was more frequently found in the subgroup with macrometastases (12/17 cases). No relationship was observed between BVI and the presence of metastases (p = 0.81).

Tumors with LVI showed higher histologic grade (p = 0.013), an increased Ki67 score (p = 0.00013), and high mitotic score (p = 0.0002), but lower estrogen (p = 0.0016) and lower progesterone (p = 0.00017) receptor scores. The other variables (p53, Her2/*neu *and E-cadherin) did not show any relation to LVI and/or BVI and lymph node status (Tables 2 and 3).

Since several of these variables are correlated, we used a logistic regression analysis with the lymphatic invasion as the dependent variable and the aforementioned variables as the independent ones, including the histologic grade. The following variables entered the final regression model, which classified 82.93% of the cases correctly: progesterone score (B=-0.3295; p = 0.0283) and Ki67 score (B = 0.7184; p = 0.0055). In contrast, we could not find a significant logistic regression model for BVI regarding the aforementioned variables.

No relationship was observed between age of patients, histologic tumor type and LVI or BVI (p > 0.05). BVI was positively related to tumor size (p = 0.009), and was more frequent in tumors greater than 2 cm. No relationship was observed between LVI and tumor size.

In addition to lymphatic vessel endothelium, the D2-40 antibody also stained myoepithelial cells of normal ducts and lobules in the breast parenchyma adjacent to the tumor in all 123 cases.

## Discussion

Our study confirmed that D2-40 stains the endothelium of lymphatic vessels and is useful and reliable in detecting LVI in invasive mammary carcinomas. In our study, LVI was related to breast cancers with high aggressive features (high Ki67 score, high histologic grade, negative hormone receptors expression). We found an inverse correlation between ER and PR expression and LVI. Similar to our study, other authors found a correlation between high histologic grade and Ki67 score to LVI, and a significantly increased risk for tumor recurrence or deaths regardless of axillary node status [3,16,35]. Instead of being explained by stroma retraction artifacts, as proposed by Acs *et al *[36], these correlations could be explained by the biologic properties of the tumor. Tumors with LVI are less differentiated, fast-growing and high grade tumors that offer greater clonal variety of tumor cells. Therefore, there is greater chance of cells capable of invading lymphatic vessels compared with low-grade, slow-growing tumors [3]. The pathophysiology of tumor related blood vessels is completely different. Capillaries are feeder vessels that ensure tissue viability, and promote tumor growth and nutrition in malignant tumors. Lymphatic vessels, on the other hand, are draining vessels that are not essential for tumor metabolism and therefore would not provide any advantage for tumor growth or survival [3,15]. Tumor vasculature has a markedly different phenotype from normal vessels, and it is highly likely that tumor lymphatic vessels also differ significantly from normal. Gene array studies on lymphatic endothelium isolated from tumors will therefore be of major interest to help develop new markers relevant to tumor therapy and outcome [27].

E-cadherin, an integral part of adherens junction, maintains the integrity of epithelial cells by facilitating cell-cell adhesion [32]. Decreased expression of E-cadherin in invasive ductal carcinomas has been correlated with higher histologic tumor grade [37,38]. We found no relationship between E-cad expression and LVI and BVI, as well as histologic grade and type and other immunohistochemical features of the primary tumor (ER, PR, Ki67, p53, and Her2/*neu *expressions), confirming results of other studies [39].

To our knowledge, this is the first study that has assessed LVI in breast cancer evaluating cases stratified in subgroups according to axillary lymph node status. LVI was positively related to status of axillary lymph node. LVI detected by both H&E and IHC was superior in the subgroup with macrometastases (39% by IHC and 29.3% by H&E), compared with the other subgroups. Our results show that LVI detected by D2-40 staining can more reliably predict lymph node metastases than the H&E-stained sections, and could be a reliable tool in predicting poor prognosis, mainly in patients with macrometastases [36].

Our results demonstrated that lymphatic and blood neoplastic emboli were found more frequently in the immunostained sections than in the H&E-stained sections. The D2-40 is superior to H&E in identifying LVI in invasive mammary carcinomas and can be used in addition to H&E in the assessment of LVI with increased accuracy [5,20,36,40]. The Kappa score obtained in our study showed only moderate agreement regarding the LVI, when comparing the H&E and immunohistochemical methods. The increased accuracy of LVI detection using IHC was previously demonstrated in breast cancer [3,5,13,16,20] and in other tumors, such as melanoma [41] and gastric cancer [4].

It has long been suggested that LVI, as a diagnostic reproducible feature, should be considered outside the tumor margin [3,13]. Yamauchi *et al *examined intratumoral, peritumoral and tumor emboli distant from tumoral areas, and despite the localization of the emboli they recommend a combination of H&E and D2-40 staining if pathologists find tumor nests suspicious of being emboli in H&E [16]. Peritumoral vascular invasion, especially lymph vascular invasion, has been included as an adverse prognostic factor in the guidelines and recommendations for postoperative adjuvant systemic therapies of early breast cancer by the International Consensus Panel during St Gallen Conference, 2005 [42]. The presence of peritumoral vascular invasion defined an intermediate risk for patients with node-negative breast disease, but its value in patients with node-positive breast disease was considered uncertain and insufficient at that time [27].

Our results are in agreement with a previous study that compared D2-40 and podoplanin on paraffin sections of a series of head and neck squamous cell carcinomas. Both antibodies were shown to have extremely high specificity (99.7% and 98.8% for podoplanin and D2-40) and sensitivity (92.6% and 97.3% for podoplanin and D2-40) for lymphatic endothelium [19]. A comparison of different endothelial lymphatic markers showed that the sensitivity of D2-40 to recognize intratumoral lymphatic vessels on serial sections of breast carcinomas is higher than that of LYVE-1, podoplanin or Prox-1 [20].

In our series, the D2-40 also stained myoepithelial cells of normal ducts and lobules of the adjacent peritumoral parenchyma. Although the antibody selectively stained the lymphatic vessel endothelium and was negative in blood vessel endothelium, it is not a specific lymphatic marker. Besides myoepithelial cells of human breast [15], it has been observed that D2-40 also stains basal epithelial cell layers of the epidermis [41] and prostate gland [43].

## Conclusion

Our results show that D2-40 is a useful tool for identification of LVI in breast carcinomas. LVI is related to tumors with higher aggressive features reflecting a potential for lymphatic metastatic spread and possible poor prognosis.

## Competing interests

The author(s) declare that they have no competing interests.

## Authors' contributions

VFZM carried out all immunostains, analyzed all H&E and IHC stained slides, analyzed lymphatic and blood vessel invasion and wrote the manuscript.

KM carried out the statistical analysis and critically reviewed the manuscript.

FSFS helped the case selection, data collecting and helped the Epi-Info analysis.

GFSR provided technical assistance and performed sections for immunostains and H&E.

HG has been involved in the study design, drafting and reviewing the manuscript critically.

All authors read and approved the final manuscript.

## Pre-publication history

The pre-publication history for this paper can be accessed here:


